# A Comprehensive In Vitro and In Silico Approach for Targeting 4-Hydroxyphenyl Pyruvate Dioxygenase: Towards New Therapeutics for Alkaptonuria

**DOI:** 10.3390/ijms26073181

**Published:** 2025-03-29

**Authors:** Giulia Bernardini, Alfonso Trezza, Elena Petricci, Giulia Romagnoli, Demetra Zambardino, Fabrizio Manetti, Daniela Braconi, Michela Geminiani, Annalisa Santucci

**Affiliations:** 1 ONE-HEALTH Lab, Department of Biotechnology, Chemistry and Pharmacy, University of Siena, Via Aldo Moro, 53100 Siena, Italy; giulia.bernardini@unisi.it (G.B.); alfonso.trezza2@unisi.it (A.T.); elena.petricci@unisi.it (E.P.); giulia.romagnoli2@unisi.it (G.R.); d.zambardino@student.unisi.it (D.Z.); daniela.braconi@unisi.it (D.B.); annalisa.santucci@unisi.it (A.S.); 2 MetabERN, Department of Biotechnology, Chemistry and Pharmacy, University of Siena, Via Aldo Moro, 53100 Siena, Italy

**Keywords:** AKU, NTBC, 4-HPPD, inhibitors, residence time, molecular modeling, docking simulation, molecular dynamics simulation, steered molecular dynamics (SMD), IC_50_, IC_90_

## Abstract

Alkaptonuria (AKU) is an ultra-rare genetic disorder caused by mutations in the homogentisate 1,2-dioxygenase (*HGD*) gene, leading to the accumulation of homogentisic acid (HGA). Current treatment options are limited, with Nitisinone (Orfadin or NTBC) being the only approved drug. However, its long-term use raises concerns due to significant adverse effects, highlighting the urgent need for safer alternatives. AKU manifests with progressive and often painful symptoms, severely impacting patients’ quality of life. Identifying new therapeutic approaches to inhibit 4-hydroxyphenyl pyruvate dioxygenase (4-HPPD) is critical to improving outcomes for AKU patients. In this study, we present a novel integrated in vitro and in silico strategy to assess the residence time of 4-HPPD inhibitors. In particular, we evaluated several features of a set of triketone compounds including their inhibitory efficacy, residence time, and ochronotic pigment accumulation. By means of our integrated approach, we investigated the pharmacokinetic and pharmacodynamics properties of novel 4-HPPD inhibitors and provided a promising foundation for the development of safer and more effective treatments for AKU.

## 1. Introduction

AKU is an ultra-rare, autosomal recessive disorder characterized by a deficient homogentisate 1,2-dioxygenase (*HGD*) enzyme activity, which is crucial in the catabolism of tyrosine and phenylalanine [1]. Mutations in the *HGD* gene lead to the accumulation of homogentisic acid (HGA), which is in part excreted in the urine (darkening upon exposure to air or alkalinization), in part progressively accumulated in connective tissues (ochronosis) [2]. Several highly debilitating symptoms are associated with HGA accumulation and ochronosis, including a severe, early-onset form of osteoarthropathy, degenerative joint disease, and cardiovascular complications, significantly impacting the quality of life of affected individuals [2,3]. Though AKU is generally considered to not impact life expectancy, there are reports of end-stage renal disease leading to untreatable, fatal acute haemolysis and/or methemoglobinemia [2].

Limited awareness of AKU, along with the non-specific nature and variability of symptoms, might lead to misdiagnosis or late diagnosis. Furthermore, there are no apparent genotype–phenotype relationships. The clinical complexity of AKU, whose molecular mechanisms are not fully elucidated yet, therefore requires early diagnosis and intervention, possibly with tailored approaches allowing both the correction of metabolic dysregulation and symptomatic relief [4].

Being an inhibitor of 4-HPPD, an upstream enzyme compared to HGD in the catabolic pathway of tyrosine [5], nitisinone (NTBC) became the first approved drug for AKU thanks to its efficacy in lowering HGA levels in affected individuals [6].

4-HPPD (EC 1.13.11.27) plays a pivotal role in converting 4-hydroxyphenylpyruvate (4-HPP) into HGA through a dioxygenation reaction [7]. This enzyme is a member of the non-heme iron-dependent dioxygenase family, featuring a distinct three-dimensional structure that typically includes a ferrous iron center essential for activity [8]. Crystallographic studies revealed that 4-HPPD forms a homodimer structure, with both monomers contributing to the active site responsible for catalytic activity [9]. The geometry of the active site is finely tuned to facilitate the interaction with 4-HPP and molecular oxygen, which is critical for the efficiency of the enzymatic process [10].

4-HPPD is predominantly expressed in the liver and kidney [11], its expression being variable among individuals and regulated by various factors, such as, dietary intake of phenylalanine and tyrosine [12]. Understanding the expression patterns of 4-HPPD is crucial, as variations may influence the metabolic fate of these amino acids and the overall production of HGA, especially in individuals with genetic conditions such as AKU [13].

After a 4-year clinical trial indicating that NTBC could reduce urinary and serum HGA, in 2020 the European Medicines Agency approved NTBC for the treatment of AKU [6,14]. It was also noted that a slower rate of disease progression could be observed in NTBC-treated subjects.

Despite therapeutic efficacy, however, the use of NTBC is not without drawbacks [15]. The consequent hypertyrosinemia is associated with ocular complications, leukopenia, and thrombocytopenia, possibly requiring drug discontinuation [16]. Therefore, there is an interest in developing novel 4-HPPD inhibitors with fewer side effects or adjunct therapies that may complement NTBC for the treatment of AKU [2]. A large number of small molecules able to inhibit 4-HPPD were reported in the last decades, mostly developed and approved as herbicides [17,18], with a few (mostly NTBC analogues) also tested for application in AKU [19]. Given the critical role of 4-HPPD in broader metabolic processes, targeting this enzyme offers a promising avenue for new treatment strategies [20]. Innovative research is underway to identify compounds that may provide additional benefits, enhancing the management of AKU and addressing the unmet medical needs of affected individuals [2].

A crucial parameter in the drug discovery process is the evaluation of residence time, defined as the duration a drug remains bound to its target enzyme [21]. It provides a dynamic measure of drug–target interactions, emphasizing the importance of sustained binding for prolonged therapeutic effects [22]. For 4-HPPD inhibitors, enhanced residence time may correlate with prolonged suppression of HGA levels, thereby reducing disease symptoms over time [5]. Therefore, understanding the kinetics of 4-HPPD interactions with various inhibitors is essential for optimizing therapeutic strategies [5].

Additionally, molecular docking studies may facilitate the identification of potential inhibitors by simulating their binding interactions with 4-HPPD, predicting binding affinities and mechanisms of action [20,23]. Furthermore, molecular dynamics (MD) simulations provide insights into the stability and dynamics of inhibitor–enzyme interactions, elucidating the molecular mechanisms that underpin inhibitor efficacy [24]. Techniques like steered molecular dynamics (SMD) can simulate the unbinding process of inhibitors, offering valuable data on binding strength and stability, which are directly related to residence time and clinical efficacy [25].

In this study, we evaluated a panel of seven 4-HPPD inhibitors, including commercially available and newly synthesized NTBC analogues, through a comprehensive and integrated in vitro and in silico approach (Scheme S1). Key parameters such as IC_50_, IC_90_, residence time, and ochronotic pigment accumulation were measured, while computational techniques were used to gain insights into the interactions with the target enzyme. This approach could represent an advancement in the search for novel and safer therapeutic options for AKU.

## 2. Results

### 2.1. In Vitro Results

#### 2.1.1. IC_50_ and IC_90_ Evaluation

**Figure 1:**
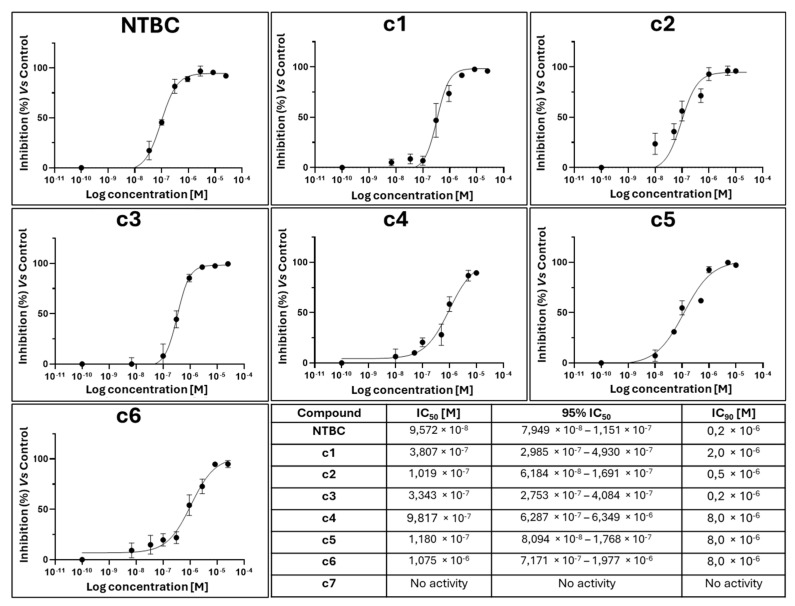
Inhibition curves obtained by testing different concentrations of the 4-HPPD inhibitors with corresponding IC_50_ and IC_90_ values.

Inhibition assays were set up to determine the IC_50_ and IC_90_ of NTBC and the novel 4-HPPD inhibitors tested in this work (c1–c7). To do so, an inhibition assay was set up to test the compounds at different concentrations. The bacterial suspension was incubated in a medium containing 1 mM IPTG, 75 µM Tyr, and the tested inhibitor, monitoring the formation of the ochronotic pigment at 405 nm after 24 h. The inhibition curves obtained for each compound are reported in Figure 1.

#### 2.1.2. 4-HPPD Inhibitor Residence Time Evaluation

**Figure 2:**
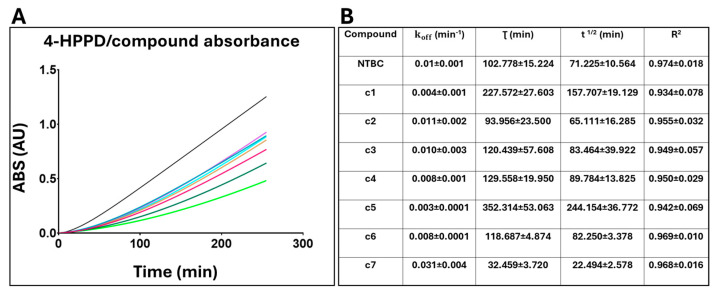
4-HPPD/compound residence time evaluation. (**A**) Enzyme activity evaluated in presence of each compound. (**B**) Details of the tested compounds with their corresponding residence time (τ) and half-life values. Magenta: NTBC; dark green: c1; turquoise: c2; purple: c3; ocher: c4; light green: c5; light blue: c6; black: c7.

To evaluate and compare the residence time (τ) of NTBC and compounds c1–c7, we considered the $k_{off}$ parameter, from which both τ and the compound half-life were derived. 4-HPPD was initially incubated with each compound at saturating concentrations, and the exhaust medium (containing the inhibitor) was removed and replaced with fresh medium without the inhibitor, while the precursor (tyrosine) was added to the substrate. Enzyme activity was monitored by measuring the formation of ochronotic pigment consequent to the formation of HGA. Each inhibitor was analyzed in duplicate, and the analyses were repeated at least three times. In each experiment, control tests were set up as follows: a blank (control without induction), a control without inhibition, and a control with inhibition. From the absorbance results, we observed that the absorbance of the inhibited control did not increase over time, as NTBC was present at an inhibitory concentration of 90%, whereas the non-inhibited control showed increasing absorbance values over time due to the absence of NTBC and the presence of the substrate precursor (Figure S1). Then, the compounds were analyzed, and a non-linear regression was performed, obtaining τ and half-life (Figure 2A,B).

Remarkably, c1, c3, c4, c5, and c6 exhibited a lower $k_{off}$ and a higher residence time value than NTBC, suggesting a more gradual dissociation from 4-HPPD. To further support our evidence, we evaluated the compound half-life, which showed that the τ^1/2^ of NTBC was lower than c1, c3, c4, c5, and c6, confirming the slower dissociation of the analogues from 4-HPPD compared to NTBC.

### 2.2. In Silico Results

#### 2.2.1. 4-HPPD Virtual Screening

Autodock/VinaXB performed docking simulation of NTBC and compounds c1–c7 on 4-HPPD. We found that all the compounds could bind to the same target binding pocket with a similar binding pose (Figure S2) and exhibited high binding free energy values (from −9.4 kcal/mol to −5 kcal/mol) (Table 1). A wide polar and hydrophobic interaction network with the side chains of residues in the 4-HPPD binding pocket could be identified (Table 1). Noticeably, all the tested compounds formed at least one hydrophobic interaction with binding pocket residues and a π-cationic interaction between the bi/triketone group oxygen and the iron ion. The analysis of the crystal structure of 4-HPPD in complex with NTBC showed very similar interactions with the residues involved in the binding compared to compounds c1–c7.

#### 2.2.2. 4-HPPD/Compound Complex Binding Stability and Interaction Energy

A total of 100 ns classical Molecular Dynamics (cMD) simulations were performed to evaluate both the stability of protein backbone and the docked poses previously selected. To exclude the presence of artifacts, the protein backbone structural integrity was monitored during the simulations. We noticed limited differences of the Root-Mean-Square Deviation (RMSD) for all systems (1.8 Å and 2.7 Å, respectively), which excluded structural rearrangements, thus corroborating the reliability of MDs protocols (Table S1). Furthermore, all compounds within target binding pocket showed very limited movements, exhibiting an RMSD average between 0.8 Å and 2.5 Å, respectively, confirming the binding stability and the reliability of docked poses (Table S1). The target/compound interaction energy was evaluated considering the Coulomb and Lennard–Jones short range interactions. This analysis showed that all the tested compounds had interaction energy values ranging from −83.4 kJ/mol to −428.4 kJ/mol, suggesting a spontaneous binding to the target with high affinity and stability.

#### 2.2.3. Compound Protein Unbinding Simulations

**Figure 3:**
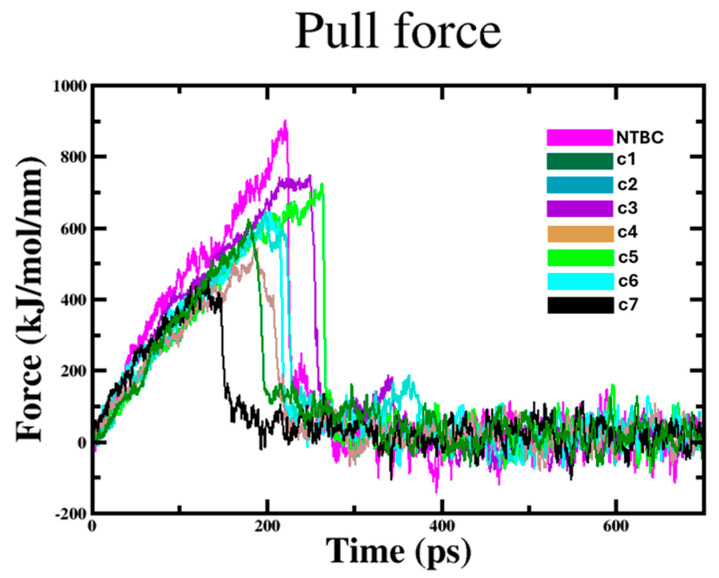
SMD simulations. Force profiles of compounds pulled out of the 4-HPPD binding pocket along the unbinding pathway.

To further dissect the unbinding process, a SMD simulation was performed on each 4-HPPD/ligand complex, reporting the time-averaged pull force profiles during the unbinding simulation. The compounds showed a gradual increase of the applied forces on the first ~180 ps and ~250 ps of the simulation, until they reached a maximum, which corresponds to the rupture force of compounds unbinding along the dissociation pathway. The quick decrease of force of each compound represents the steady path of the ligand moving away from the target binding pocket; the unbinding process proceeds until the end of the simulation. Surprisingly, despite the good binding stability and the highest interaction energy score of c7 on the target, SMD results showed that c7 (no active compound) was the first compound to leave the binding pocket; differently, c3 and c5 (compounds with highest activity) were the last to dissociate from the target, in line with the experimental data, as well as the force profiles of other compounds (Figure 3).

## 3. Discussion

The aim of this study was to investigate the residence time of novel 4-HPPD inhibitors, using both experimental and computational approaches, and provide a comprehensive understanding of ligand–receptor interactions. NTBC, currently approved for the treatment of AKU and HT-1, can effectively inhibit 4-HPPD, but it is associated with significant side effects consequent to the induced hypertyrosinemia. Therefore, safer but effective therapeutic options are needed for AKU patients, whose life expectancy is generally not affected by the disease and hence require life-long treatment with NTBC.

In our study, the evaluation of the residence time (τ) complements traditional affinity metrics such as IC_50_ and IC_90_, providing deeper insights into the pharmacodynamics of drug–target interactions, important in the evaluation of both efficacy and therapeutic outcomes.

An optimized version of the jump dilution method was employed to evaluate the residence time of the novel candidate 4-HPPD inhibitors. Specifically, a recombinant strain of *E. coli* expressing human 4-HPPD was used as a cellular model, enabling real-time kinetic monitoring of the enzyme’s activity through UV-Vis spectroscopy. This approach allowed precise tracking of reaction kinetics with high temporal resolution. After determining the IC_50_ and IC_90_ values for each tested compound, the enzyme was incubated with the inhibitors at concentrations corresponding to IC_90_. The exhaust medium containing the inhibitor was subsequently replaced with a fresh and inhibitor-free medium supplemented with tyrosine, the substrate precursor. The recovery of enzymatic activity was monitored over time using spectrophotometric analysis. This analytical approach provided precise measurements of the residence time for each inhibitor. The results showed that six of the seven tested compounds exhibited longer residence time than NTBC, indicating a potentially greater inhibitory effect.

Computational analyses were conducted to further investigate the structure–activity relationship (SAR) of these inhibitors. Molecular docking studies demonstrated that all compounds bound spontaneously to the 4-HPPD active site with high affinity, adopting similar binding poses and interaction networks compared to NTBC. To further explore the potential mechanism of action, cMD simulations were performed. These simulations provided insights into the dynamic behavior of the enzyme–ligand complexes under biologically relevant conditions, elucidating the stability and binding efficiency of each compound.

Additional computational analyses, including RMSD and interaction energy calculations, did not reveal significant differences in conformational changes or interaction energies between the compounds along the entire MD run. This suggested that variations in inhibitory activity might not be solely related to pharmacodynamics or enzyme conformational shifts. The data indicated that 4-HPPD inhibition was influenced by other factors, including the interaction with the Fe(II) ion in the active site. This interaction appeared to be correlated with an increased residence time, which in turn enhanced the inhibitory effect.

The computational study provided valuable insights into the un/binding processes of new NTBC analogues with 4-HPPD, with SMD results in strong agreement with the experimental findings. All inhibitors were bound in active sites that were partially blocked by the C-terminal α helix. In particular, the two carbonyl groups (except for c7) of the inhibitors were conjugated with the metal ion, which is chelated by the conserved facial triad consisting of His183, His266, and Glu349. Additionally, the phenyl moiety of the inhibitors forms pronounced π–π stacking interactions with His226, His308, Phe336, and Phe364, and the cyclohexane moiety shows a wide hydrophobic network (Table 1, and Figure S3). Interestingly, all inhibitors except NTBC and c1 formed one or more hydrogen bonds within the target binding pocket as well as the salt bridge for c5 and c6 with His266, while only c6 and c7 formed a halogen bond with Phe359 and Glu360. Based on the known and strong inhibitory activity of NTBC against 4-HPPD, as well as the inhibitors proposed by us proposed in this study and their interaction network, we support the idea of the critical role of metal ion coordination in increasing the ligand residence time, contributing to a better understanding of the molecular basis of inhibition; further, considering the large difference of the chemical–physical properties of compounds, several pharmacokinetics insights were provided.

## 4. Materials and Methods

### 4.1. In Vitro Methods

#### 4.1.1. Bacteria Cell Culture

The pre-inoculum was prepared in LB medium (Lysogeny Broth) supplemented with glucose (1% final concentration) and kanamycin sulfate (50 µg/mL final concentration). A small quantity of bacterial cells was taken from the frozen stock of recombinant *E. coli* C43 (DE3) expressing human 4-HPPD in 40% glycerol using a sterile loop and added to the prepared LB medium (LBG + antibiotic). The pre-inoculum was incubated overnight at 37 °C under agitation. After measuring the OD_600_ of the pre-inoculum using LB medium as a blank, it was diluted to a final optical density of 0.1 in LB medium supplemented with kanamycin (LBK). The cell culture was shaken at 37 °C, and the OD_600_ was measured every 45 min until reaching the mid-log phase (0.6–0.8). Protein expression was then induced by adding isopropyl-β-D-thiogalactopyranoside (IPTG) [26,27,28].

#### 4.1.2. Chemistry

NTBC, mesotrione (c1), tembotrione (c2), and Sulcotrione (c3) were already available in our laboratory. 5,5-dimethylcyclohexane-2-(2-chloro-4-nitrobenzoyl)-1,3-dione (c4), 5,5-dimethyl-2-(2-nitro-4-(trifluoromethyl)benzoyl)cyclohexane-1,3-dione (c5), 2-(2-chloro-4-nitrobenzoyl)1,3-cyclohexanedione (c6), and 5,5-dimethyl-3-oxocyclohexenyl 2-chloro-4-(trifluoromethyl) benzoate (c7) have been newly synthesized in our lab. The characterization of NTBC and compounds c4–7 is reported in Supporting Information (Figure S4).

#### 4.1.3. IC_50_ and IC_90_ Evaluation

To calculate the 50% (IC_50_) and 90% (IC_90_) inhibitory concentrations of each compound, an inhibition assay was set up in which varying concentrations of the inhibitors were tested [27,28]. Briefly, serial dilutions of the inhibitors were prepared in 96-deep well standard plates, starting from a stock solution of 10^−2^ M, and then 600 µL of bacterial suspension (see Section 4.1.1) supplemented with 1 mM IPTG and 75 µg/mL Tyr were added into each well.

In parallel, the control tests were set up as follows:

- Blank: culture medium without cell suspension.
- Negative control: cell suspension without IPTG and Tyr.
- Not-Inhibited control: cell suspension supplemented with 1 mM IPTG and 75 µg/mL Tyr.

The plate was incubated on a microplate shaker at 800 rpm for 24 h.

The plates were centrifuged at 3200× *g* for 10 min at 4 °C. Subsequently, 150 μL of supernatant (pigmented medium) was transferred to a 96-well standard plate, and the absorbance was measured at 405 nm using a multimode plate reader (EnVision 2105, PerkinElmer Inc., Waltham, MA, USA). GraphPad Prism 8 was used to perform a nonlinear regression analysis (Log[Inhibitor] vs. response—Variable slope (four parameters)) and to obtain the inhibition curve of the compounds.

#### 4.1.4. Residence Time Evaluation

The residence time of potential 4-HPPD inhibitors was evaluated using an experimental protocol developed in-house, based on the classic jump dilution method [29,30] and the use of recombinant *E. coli* strain expressing human 4-HPPD:

7 mL of bacterial suspension (see Section 4.1.1) at the mid-log phase was (OD_600_ = 0.6–0.8) were transferred into sterile 50 mL centrifuge tubes and experiments set up as follows:

- Blank: cell suspension without the IPTG.
- Positive control: cell suspension supplemented with 1 mM IPTG.
- Negative control: cell suspension supplemented with 1 mM IPTG and NTBC (0.2 µM final concentration).

In addition to 1 mM IPTG, potential inhibitors (at the final IC_90_ concentration) were added to the other tubes. Each test was performed in duplicate, and the experiments were replicated at least three times. The tubes were half-capped and stirred at room temperature overnight.

The tubes were centrifuged at 2500× *g* for 10 min at 20 °C. The supernatant (exhaust medium) was discarded, while the pellet was resuspended in 7 mL of fresh LBK medium. Tyrosine (75 µg/mL) was then added to each tube (excluding the blank), and NTBC (0.2 µM final concentration) was also added to the negative control to maintain an inhibitory concentration of 90%.

The tubes were incubated at 37 °C with stirring after taking the first sample (T_0_). Then, every 45 min for a period of 8 h, 220 μL were taken from each tube and placed in an Eppendorf tube, which was centrifuged at 13,000× *g* for 5 min at 4 °C. Subsequently, 200 μL of supernatant (pigmented medium) from each Eppendorf tube was transferred to a 96-well standard plate, and the absorbance at 405 nm was measured using a multimode plate reader (EnVision 2105, PerkinElmer Inc., Waltham, MA, USA).

Linear regression analysis was performed to calculate the slope values for each of the two curves corresponding to $v_{s}$ and $v_{0}$. These two parameters were then substituted into the equation below and reported for the nonlinear regression analysis of the data related to the tested compounds:

$$Y=v_{s}X+\frac{\left(v_{0}-v_{s}\right)\;}{K}\;(1-e^{-KX})$$

*Y*: Absorbance value (HGA accumulation);
*X*: time (min);
$v_{s}$: steady-state velocity;
$v_{0}:$ initial velocity;
*K*: $k_{off}$.

### 4.2. In Silico Methods

#### 4.2.1. Structural Resources

3D Structure and FASTA sequence (UniProtKB Entry: Q53586) of human 4-HPPD (4-HPPD) in complex with NTBC (PDB ID 1T47) were retrieved from the RCSB Protein Data Bank [31] and UniProt [32]. To avoid errors during the MD simulations, missing side chains and steric clashes in PDB files were adjusted through homology modelling, using PyMOD3.0 [33] and MODELLER v.9.3 [33]. 3D structures were validated using PROCHECK [34]. GROMACS 2019.3 [35] with charmm36-mar2019 force field was used to resolve high energy intramolecular interactions before docking simulations, and CGenFF 4.5 [36] was used to assign all parameters to ligands. Structures were immersed in a cubic box filled with TIP3P water molecules [37] and counter ions to balance the net charge of the system. Simulations were run applying periodic boundary conditions. The energy of the system was minimized with 5000 steps of minimization with the steepest descent algorithm and found to converge to a minimum energy with forces less than 100 kJ/mol/nm. A short 10 ns cMD was performed to relax the system. All the cMD simulations were performed integrating each time step of 2 fs; a V-rescale thermostat maintained the temperature at 300 K [38] and Berendsen barostat maintained the system pressure at 1 atm, with a low dumping of 1 ps^−1^; the LINCS algorithm constrained the bond lengths involving hydrogen atoms [39,40].

#### 4.2.2. Molecular Docking

The 3D structures of NTBC (CID: 115355), c1 (CID: 175967), c2 (CID: 11556911), and c3 (CID: 91760) were downloaded from the PubChem database [41]. The 3D structures of c4, c5, c6, and c7 (for which 2D/3D structures were not available because they were synthesized in-house) were designed and optimized using ChemDraw Professional 16.0 and downloaded in SDF format. The OpenBabel tool was used to generate the 3D structures in PDBQT format, adding all polar hydrogen atoms and assigning Gasteiger partial charges [42,43]. Virtual screening of the compounds against 4-HPPD was performed using AutoDock/VinaXB 1.1.2 [44,45]. The reaction environment was set by creating a box that encompassed all residues of the NTBC binding site, with a size of 20 × 20 × 20 Å for each dimension. The docking output generated 10 binding poses for each ligand, while all other parameters were kept at their default settings. The target/ligand interaction network was investigated using the Protein–Ligand Interaction Profiler (PLIP) tool [46,47]. The selection criteria for the best binding pose for each ligand were based on both their binding free energy values and the interaction network triggered by the target binding residues, considering the binding pose of NTBC in the 1T47 co-crystal. Each 4-HPPD/compound complex was then relaxed as described above, providing the initial conformation for molecular dynamics simulations.

#### 4.2.3. Steered Molecular Dynamics (SMD) Simulations

To evaluate the binding interactions between 4-HPPD and its ligands, the target/compound complexes were subjected to a 500 ps of SMD simulation using constant force pulling at 250 kJ/mol/nm [48]. While the backbone of 4-HPPD was kept fixed, the compounds experienced a constant force in the x (250 kJ/mol/nm), y (0 kJ/mol/nm), and z (0 kJ/mol/nm) directions. The ligands were pulled with an umbrella external force (harmonic potential) in the NPT ensemble at 1 atm and 300 K, with 2 fs time steps. Molecular dynamics (MD) analyses were performed using the GROMACS 2019.3 package [35] and visualized with GRACE 5.1.22.

## 5. Conclusions

Concluding, our work emphasizes the importance of residence time in drug discovery and highlights the added value of combining in vitro and in silico methodologies in the search of novel 4-HPPD inhibitors with potentially better profiles. Our findings might offer promising insights into the treatment of AKU, tackling the current limitations for this iconic disease and advancing the discovery of next-generation 4-HPPD inhibitors with improved safety profiles and therapeutic efficacy.

## Figures and Tables

**Table 1:** Target/compound Interaction network and binding free energy. * P = Present; * N,P = Not Present.

Compound	Hydrophobic Interaction	Halogen Bond	π-Stacking	Hydrogen Bond	Salt Bridge	Iron Ion Coordination	ΔG (kcal/mol)
NTBC	Phe-336, Phe-359, Phe-364	* N,P	* N,P	* N,P	* N,P	* P	−9.4
c1	His-226, Gln394	* N,P	His-308	* N,P	* N,P	* P	−5.0
c2	His-183, Val-185, Leu-224, Phe-359, Gly-360, Ala-361	* N,P	His-226, Phe-364	Gln-251, Gln-265, Gln-334	* N,P	* P	−8.6
c3	Leu-224, Phe-359	* N,P	Phe-336	Asn-241, Gln-265	* N,P	* P	−8.5
c4	His-183, Val-185, Leu-224, His-226, Gln-334, Phe-359, Gly-360, Ala-361	* N,P	* N,P,	Asn-241, Gln-265	* N,P	* P	−6.3
c5	Pro-239, Phe-336, Phe-359, Phe-364	* N,P	* N,P	Asn-241, Gln-251	His-266	* P	−8.0
c6	Val 185, Pro-239, Phe-336, Phe-359, Phe-364	Phe-359	Phe-364	Asn-241	His-266	* P	−8.7
c7	Leu-224, Phe-359	Gly-360	Phe-336	Asn-241, Gln-251	* N,P	* P	−7.9

## Data Availability

The original contributions presented in this study are included in the article. Further inquiries can be directed to the corresponding author.

## Supplementary Materials

The following supporting information can be downloaded at https://www.mdpi.com/article/10.3390/ijms26073181/s1.
