# Supplementary material for: A Comprehensive In Vitro and In Silico Approach for Targeting 4-Hydroxyphenyl Pyruvate Dioxygenase: Towards New Therapeutics for Alkaptonuria
Source: Int J Mol Sci. 2025 Mar 29;26(7):3181. doi: 10.3390/ijms26073181 (PMC11988800; doi:10.3390/ijms26073181)
Supplement: Supplementary file 1 [file ijms-26-03181-s001.zip › SI.pdf]

A comprehensive in vitro and in silico approach for Targeting 4-HPPD: Towards New Therapeutics for Alkaptonuria

Giulia Bernardini [1,†], Alfonso Trezza [1,†] , Elena Petricci [1], Giulia Romagnoli [1], Demetra Zambardino [1] Fabrizio Manetti [1], Daniela Braconi [1], Michela Geminiani [1,*], and Annalisa Santucci [1,2]

[1] Department of Biotechnology, Chemistry and Pharmacy, University of Siena Via Aldo Moro, Siena, 53100, Italy; giulia.bernardini@unisi.it; alfonso.trezza2@unisi.it; elena.petricci@unisi.it; giulia.romagnoli2@unisi.it; d.zambardino@student.unisi.it; daniela.braconi@unisi.it; geminiani2@unisi.it; annalisa.santucci@unisi.it

[2] MetabERN, Department of Biotechnology, Chemistry and Pharmacy, University of Siena Via Aldo Moro, Siena, 53100, Italy; annalisa.santucci@unisi.it

[*] Correspondence: geminiani2@unisi.it

[†] Contributed equally

Compound characterization

5,5-dimethylcyclohexane-2-(2-chloro-4-nitrobenzoyl)-1,3-dione (c4): ^{1}H NMR (600 MHz, $CDCl_3$, δ ppm): 16.23 (s, 1H), 8.47 (s, 1H), 7.97 (d, 1H, *J* = 9.8), 7.40 (d, 1H, *J* = 7.9), 2.67 (s, 2H), 2.24 (s, 2H), 1.11 (s, 6H). ^{13}C NMR (100 MHz, $CDCl_3$, δ ppm): 195.4, 194.1, 193.6, 145.1, 139.1, 130.3, 127.4, 123.5, 120.7, 111.4, 50.7, 44.8, 30.8, 27.7 (2C).

5,5-dimethyl-2-(2-nitro-4-(trifluoromethyl)benzoyl)cyclohexane-1,3-dione (c5): ^{1}H NMR (600 MHz, $CDCl_3$, δ ppm): 16.20 (s, 1H), 8.44 (s, 1H), 7.93 (d, 1H, *J* = 7.9 Hz), 7.37 (d, 1H, *J* = 7.4 Hz), 2.64 (s, 2H), 2.21 (s, 2H), 1.08 (s, 6H). ^{13}C NMR (100 MHz, $CDCl_3$, δ ppm): 196.08, 194.85, 194.29, 145.84, 139.17, 131.08, 131.84, 131.01, 128.10, 121.4, 112.08, 51.4, 45.5, 31.51, 28.3 (2C). ESI-MS: 358 $[M+H]^+$, 380 $[M+H]^+$.

2-(2-chloro-4-nitrobenzoyl) 1,3-cyclohexanedione (c6): ^{1}H NMR (600 MHz, $CDCl_3$, δ ppm): 8.19-8.11 (m, 1H), 8.05-8.03 (m, 1H), 7.35 (m, 1H), 4.6 (s,1H), 2.58 (s, 2H), 1.8 (s, 2H), 1.3 (s, 2H). ^{13}C NMR (151 MHz, $CDCl_3$) δ 197.17 (2C), 195.73, 165.45, 131.00, 129.75, 127.53, 124.64, 122.06, 63.03, 37.54 (2C), 19.08.

5,5-dimethyl-3-oxocyclohexenyl 2-chloro-4-(trifluoromethyl) benzoate (c7): ^{1}H NMR (600 MHz, $CDCl_3$, δ ppm): 7.99 (d, 1H, *J* = 8.02), 7.72 (s, 1H), 7.59 (d, 1H, *J* = 8.2), 6.04 (s, 1H), 2.53 (s, 2H), 2.30 (s, 2H), 1.12 (s, 6H). ESI-MS: 347 $[M+H]^+$
