# Supplementary figures and images for: A Comprehensive In Vitro and In Silico Approach for Targeting 4-Hydroxyphenyl Pyruvate Dioxygenase: Towards New Therapeutics for Alkaptonuria
Source: Int J Mol Sci. 2025 Mar 29;26(7):3181. doi: 10.3390/ijms26073181 (PMC11988800; doi:10.3390/ijms26073181)

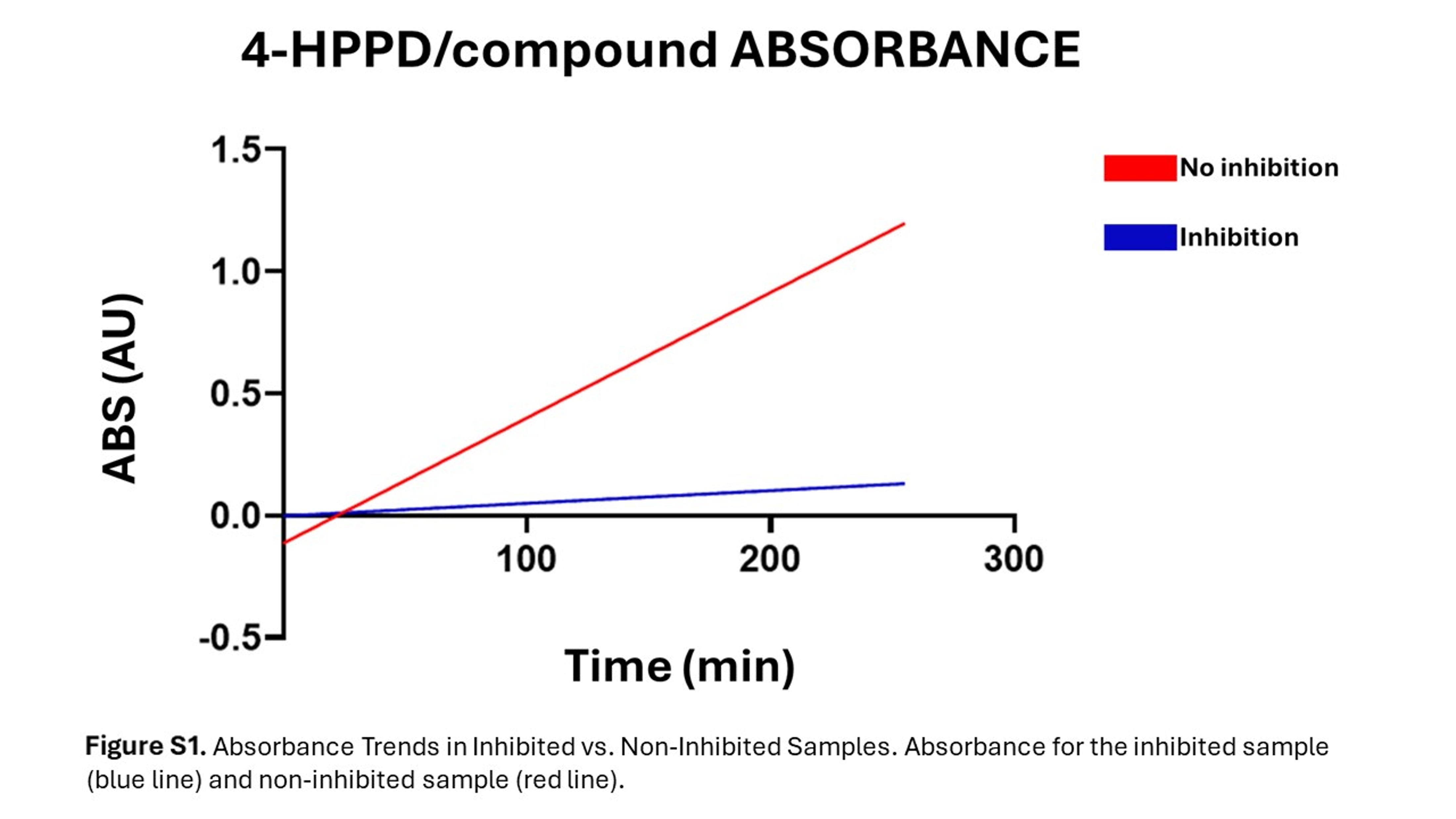

Supplement: Supplementary file 1 [file ijms-26-03181-s001.zip › Figure S1.png]

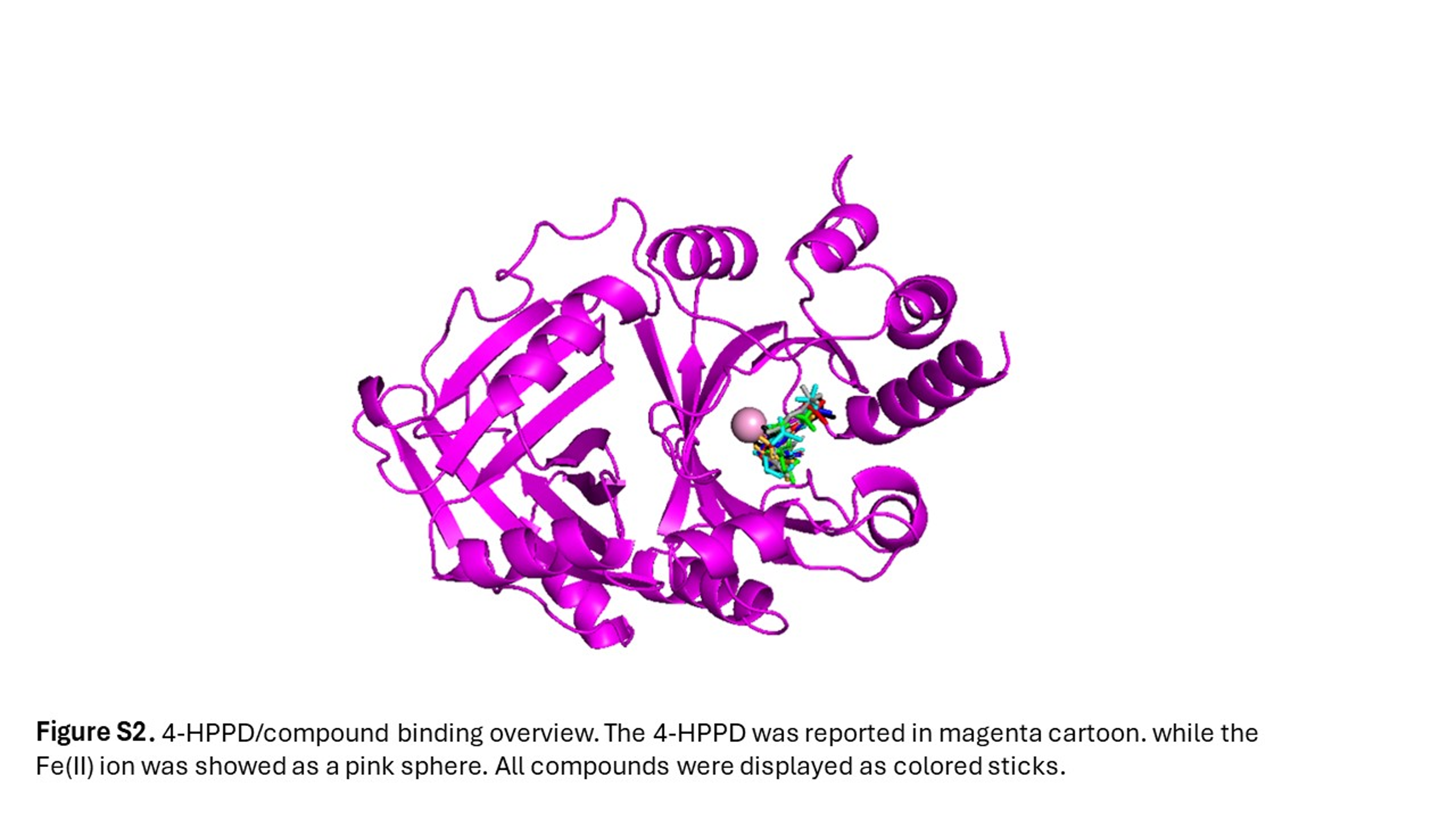

Supplement: Supplementary file 1 [file ijms-26-03181-s001.zip › Figure S2.png]

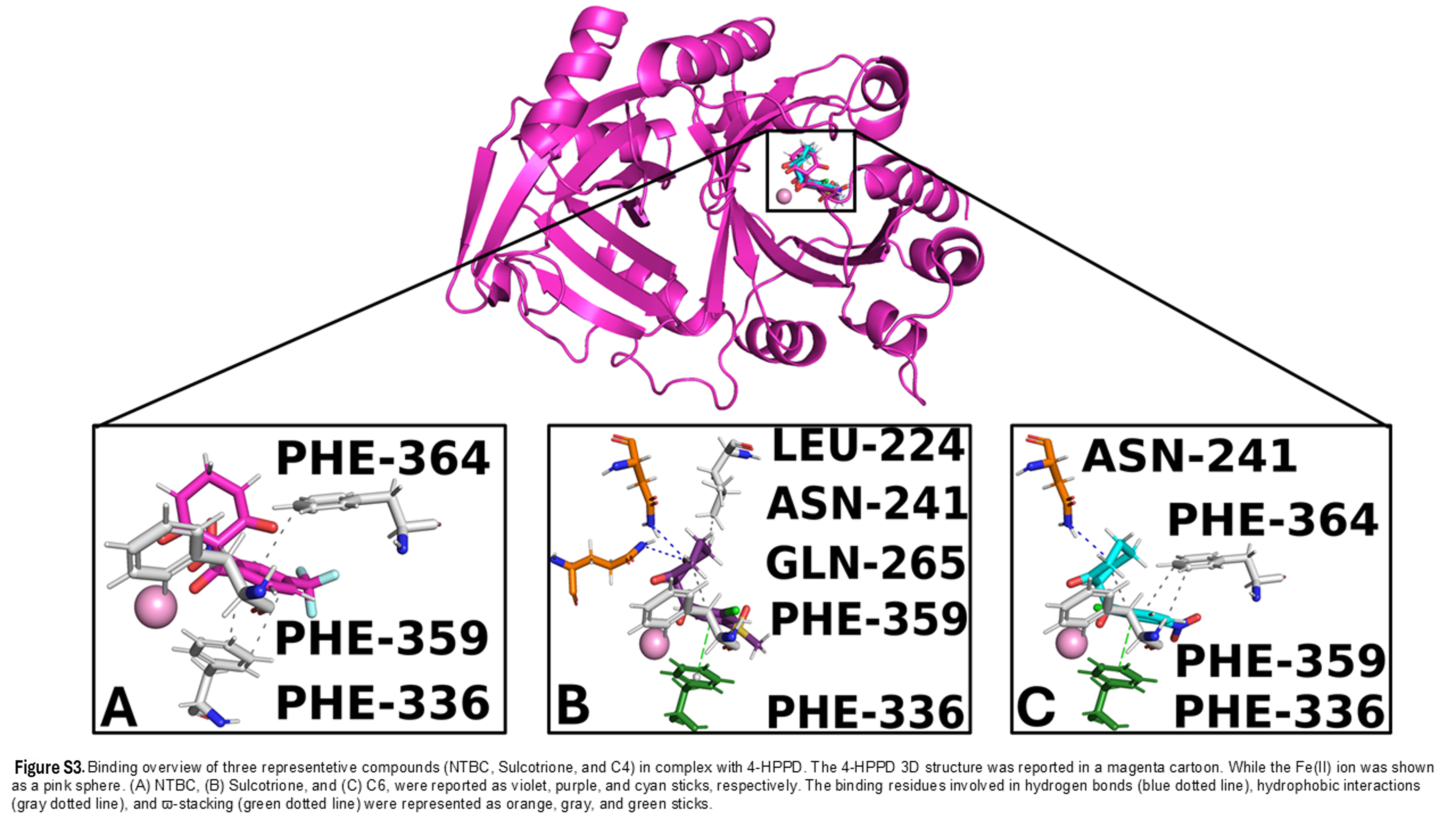

Supplement: Supplementary file 1 [file ijms-26-03181-s001.zip › Figure S3.png]

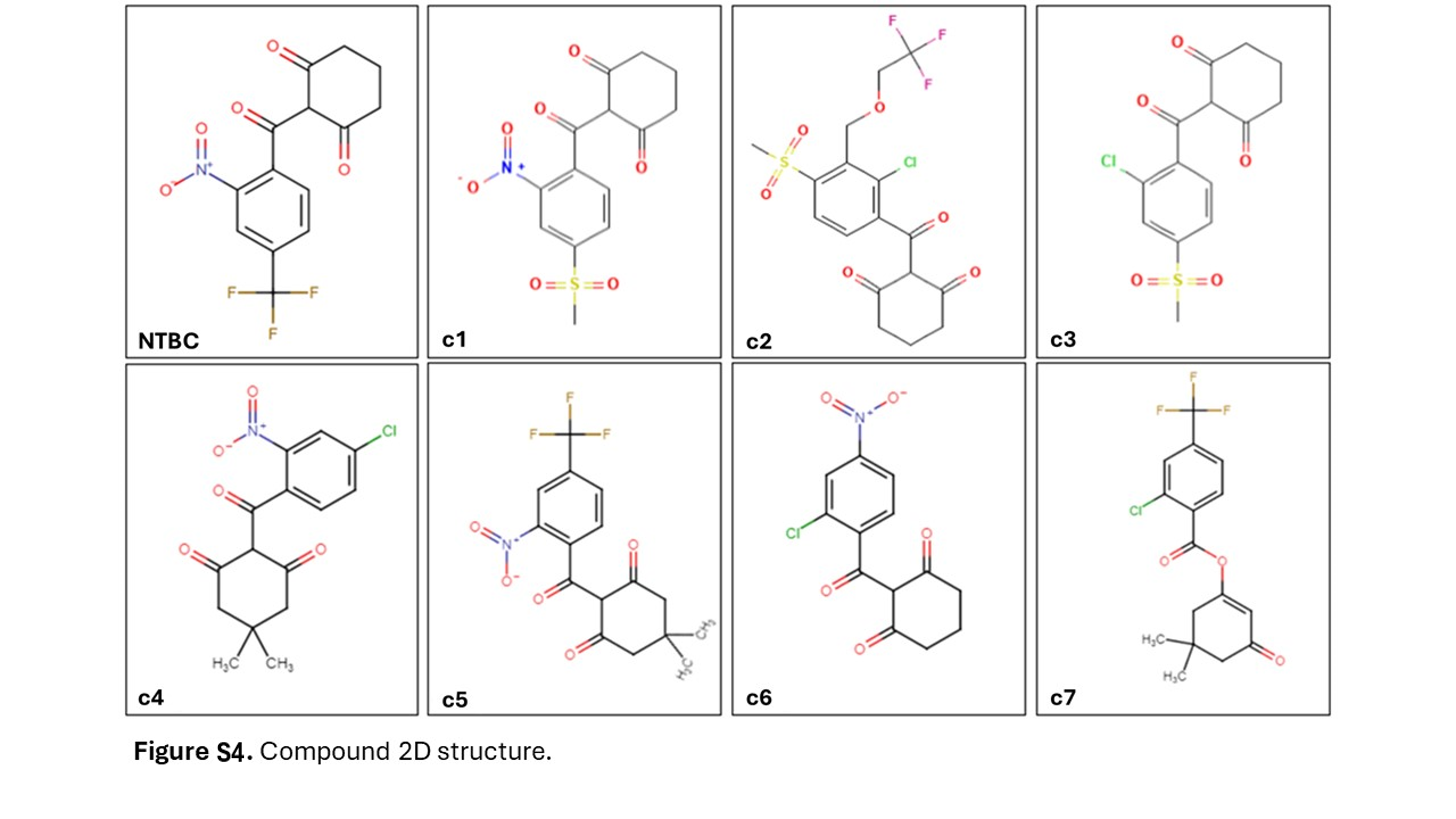

Supplement: Supplementary file 1 [file ijms-26-03181-s001.zip › Figure S4.png]

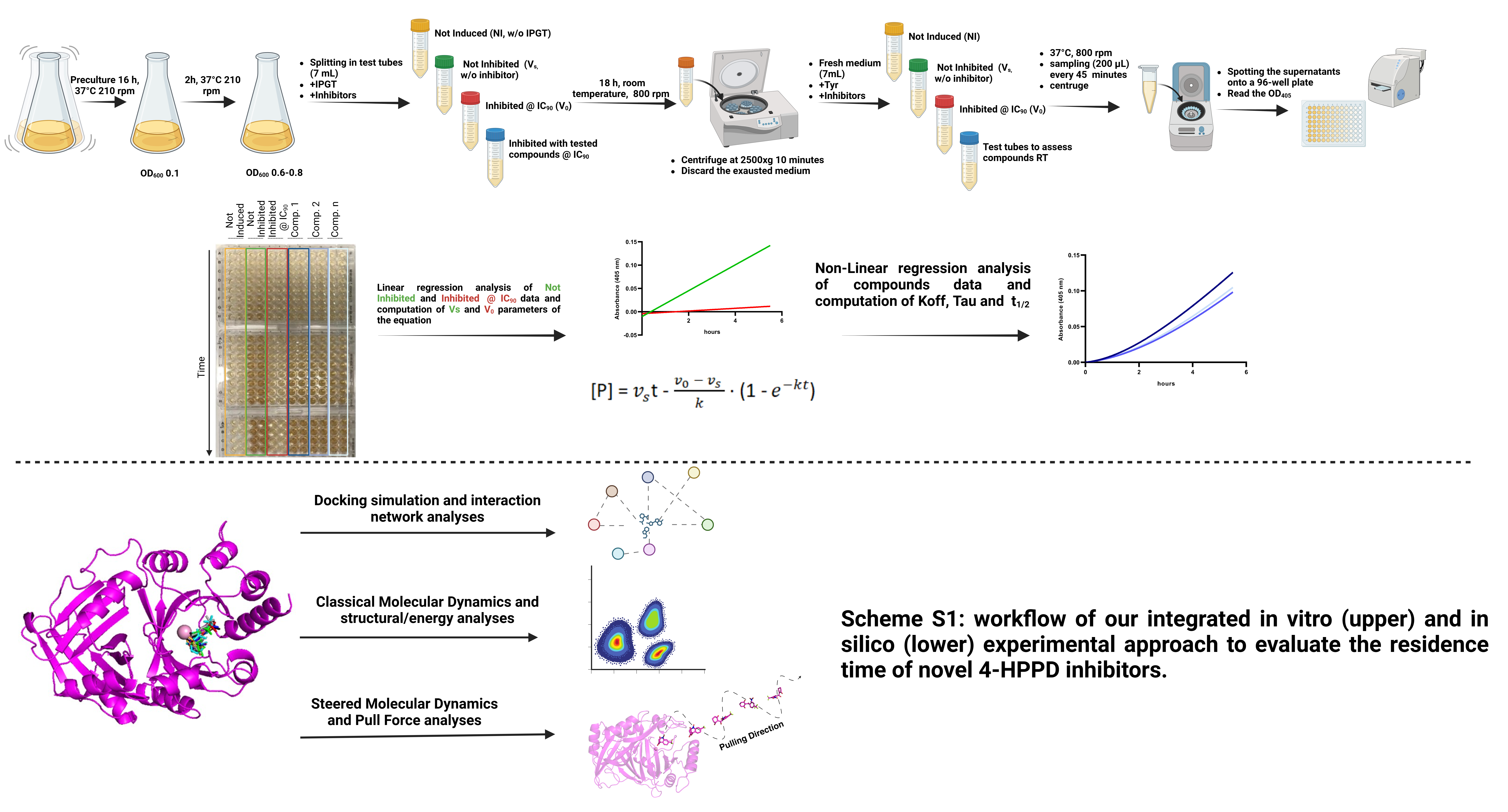

Supplement: Supplementary file 1 [file ijms-26-03181-s001.zip › Scheme S1.png]

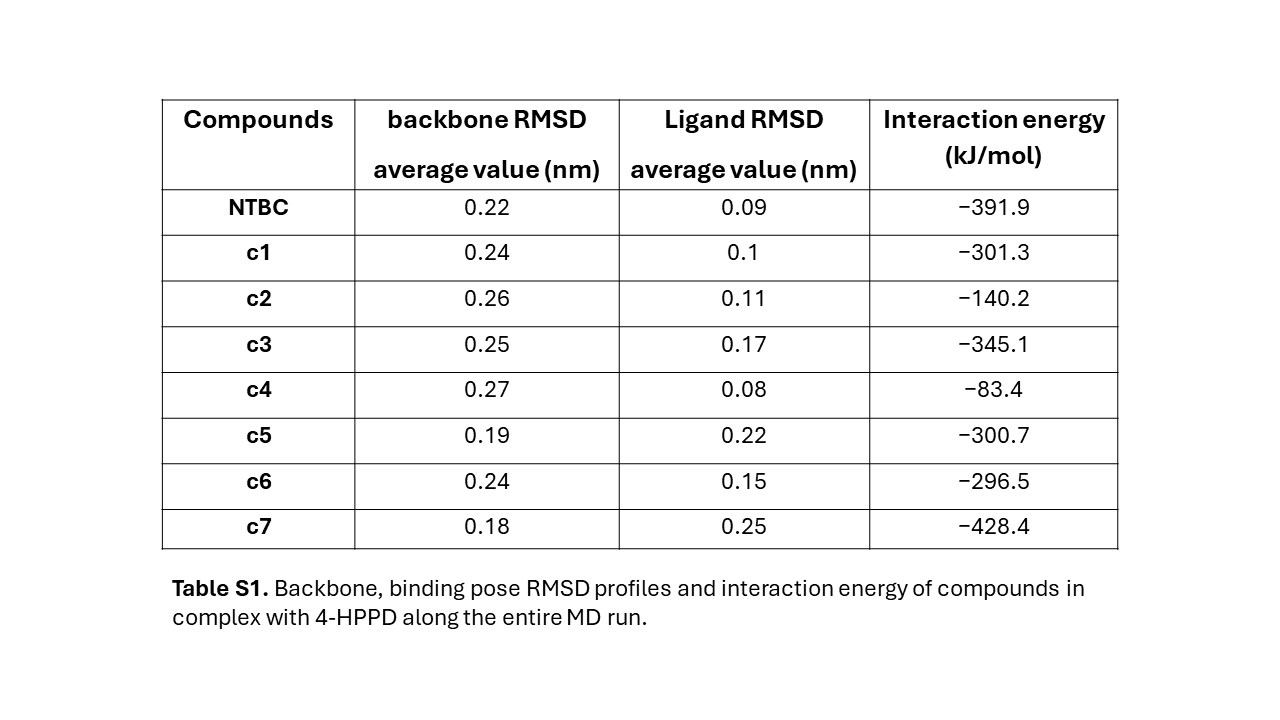

Supplement: Supplementary file 1 [file ijms-26-03181-s001.zip › Table S1.JPG]
